# Reviewer acknowledgement 2013

**DOI:** 10.1186/1472-6874-14-9

**Published:** 2014-01-17

**Authors:** Peter O'Donovan

**Affiliations:** 1 BioMed Central, 236 Gray’s Inn Road, London WC1X 8HB, UK

## Abstract

**Contributing reviewers:**

The editors of BMC Women’s Health would like to thank all our reviewers who have contributed to the journal in Volume 13 (2013).

## 

Mesganaw Fantahun Afework

Ethiopia

Sohail Agha

USA

Olufunke Alaba

South Africa

Mussie Alemayehu

Ethiopia

Richard Amenyah

Ghana

Neil Andersson

Mexico

Bengt Andrae

Sweden

Uduak Andy

USA

Uzochukwu Aniebue

Nigeria

Rose Anorlu

Nigeria

Alberto Argentiero

Italy

Benoit Arsenault

Netherlands

Erika Elizabeth Atienzo

Mexico

Jodie Avery

Australia

Pinar Ay

Turkey

Susan Ayers

UK

Rogers Ayiko

Uganda

Matthew (Mateo) Banegas

USA

Sushanta Banerjee

India

Maggi Banning

UK

Robert Barkin

USA

Mags Beksinska

South Africa

Suzanne Belton

Australia

Ebrahim Bera

South Africa

Harjit Pal Bhattoa

Hungary

Suhua Bi

China

Deborah Billings

USA

Zeev Blumenfeld

Israel

Vincent Boama

UK

Johan Bohr

Sweden

Imre Boncz

Hungary

Roslin Botlero

Australia

Marek Brabec

Czech Republic

Sharon Brennan

Australia

Benjamin Buecking

Germany

Anne Burke

USA

Jennifer Butt

South Africa

Alessandra Chacham

Brazil

Eric Chamot

USA

Sally Chan

Singapore

Venkatraman Chandra-Mouli

Switzerland

Yeun-Chung Chang

Taiwan

Jane Chiu

Taiwan

Catherine Chojenta

Australia

Cari Clark

United States Minor Outlying Islands

Anne Cockcroft

Botswana

Rachel Cooper

UK

Josip Culig

Hungary

Tienhan Sandrine Dabakuyo

France

Koustuv Dalal

Sweden

Florence Dallo

USA

Anne Kjersti Daltveit

Norway

Dharampal Dambhare

India

Edward T. Dassah

Ghana

Jokin De Irala

Spain

Harlinde De Schutter

Belgium

Singh Debra

Uganda

Beena Cr Devi

Malaysia

Dinesh Dhanwal

India

Cyril Dim

Nigeria

Guzel Discigil

Turkey

Bosiljka Djikanovic

Serbia

Priscilla Dlamini

Swaziland

Mai Do

USA

David Doku

Ghana

Alice Domar

USA

Serena Donati

Italy

Janice Du Mont

Canada

Michael Dunne

Australia

Katie Edwards

USA

Samar El Khoudary

USA

Abdellah El Maghraoui

Morocco

Nülüfer Erbil

Turkey

Magdalena Esteva

Spain

Ellen Evans

USA

Hyginus Ezegwui

Nigeria

Trevor Ferguson

Jamaica

Betsy Foxman

USA

Nicola Fratelli

Italy

Rebecca French

UK

Emilie Friberg

Sweden

Flavio Fuchs

Brazil

Julia Gage

USA

Michael Gardner

UK

Aleksandra Gentry-Maharaj

UK

Paolo Giorgi Rossi

Italy

Norman Goldstuck

South Africa

Rodolfo Gomez Ponce De Leon

Brazil

Kleanthi Gourounti

Greece

Enrique Gracia

Spain

Fredrik Granath

Sweden

Robert Grant

UK

Julie Gregory

Canada

Elfriede Greimel

Austria

Cynthia Gross

USA

Sylvia Guendelman

USA

Tamar Gur

USA

Ali Irfan Guzel

Turkey

Takashi Hajiro

Japan

Kelli Hall

USA

Jeong Yeob Han

USA

Liisa Hantsoo

USA

Sioban Harlow

USA

Hong He

China

Lotti Helström

Sweden

Stanley Henshaw

USA

Helen Herrman

Australia

Michaela Higgins

USA

N. Zoe Hilton

Canada

Raymond Hobbs

USA

Birgitta Hovelius

Sweden

Penelope Howards

USA

Ko-En Huang

Taiwan

Latifat Ibisomi

South Africa

Macit Ilkit

Turkey

Jennifer D. Irwin

Canada

Shinya Ishii

Japan

Khaled Ismail

UK

Lisa Iversen

UK

Jun Iwamoto

Japan

Hajime Iwasa

Japan

Pascal Izzicupo

Italy

Mohammed Jan

Saudi Arabia

Imke Janssen

USA

Monica Jarrett

USA

Rajesh Jeewon

Mauritius

Mona Jeffreys

UK

Carol Joinson

UK

S. M. Mostafa Kamal

Bangladesh

Bhushan Kapur

Canada

Aysun Karabulut

Turkey

Kristina Karvinen

Canada

Jennifer Katz

USA

Ravneet Kaur

India

Patrick Kayembe

Democratic Republic of the Congo

Lital Keinan-Boker

Israel

Michelle Kermode

Australia

Deborah Kim

USA

Maggie Kirkman

Australia

David Klein

USA

Stefanie Klug

Germany

Kirsten Kluivers

Netherlands

Tellervo Korhonen

Finland

Jeffrey Kromrey

USA

Abhishek Kumar

India

Manisha Kumar

India

Hann-Chorng Kuo

Taiwan

Alain Labrique

USA

Toine Lagro-Janssen

Netherlands

Maria Lahuerta

USA

Yihunie Lakew

Ethiopia

Lisa Langsetmo

Canada

Sarah Larkins

Australia

Per-Göran Larsson

Sweden

Stephen Lawoko

Sweden

Beverley Lawton

New Zealand

Mark Lazenby

USA

Andrew Levy

UK

Yanwei Li

Netherlands

Min Lian

USA

Jutta Lindert

Germany

Natalia Linos

USA

Sherry Lipsky

USA

Placido Llaneza

Spain

Sylvie Lo Fo Wong

Netherlands

Alexandre Lobo

Brazil

Riitta Luoto

Finland

Catherine Macphail

South Africa

Ferdinando Maggioni

Italy

Abdulkarim Mairiga

Nigeria

Roberto Marci

Italy

Jean Martial Mari

France

Daniela Marschall-Kehrel

Germany

Ligia Martini

Brazil

Ma. Luisa Marvan

Mexico

Mário Rui Mascarenhas

Portugal

Leslie Massad

USA

Dinos Mavromoustakis

Cyprus

Abdul Mazid

Japan

Anthony Mbonye

Uganda

Jennifer Mersereau

USA

Liselotte Mettler

Germany

Parvin Mirmiran

Iran

Hiten Mistry

UK

Ambrish Mithal

India

Emilia Montagna

Italy

Ali Montazeri

Iran

Karine Morcel

France

Daniel Mroczek

USA

Elizabeth Mueller

USA

Lars Munck

Denmark

Terra Murray

Canada

Jane Myers

USA

Nicola Nante

Iran

Rahel Nardos

USA

Christopher Naugler

Canada

Mzikazi Nduna

South Africa

Laura Nellums

UK

Dong-Young Noh

South Korea

Romana Novakovic

Serbia

Uchenna Nwagha

Nigeria

Lydia O'Donnell

USA

Akin-Tunde Odukogbe

UK

Adeola Olaitan

UK

Azubuike Onyebuchi

Nigeria

Enis Ozkaya

Turkey

Vahit Ozmen

Turkey

Benjamin Ozumba

Nigeria

Faustino Pérez-López

Spain

Rodolfo Pacagnella

Brazil

Hsiang-Chu Pai

Taiwan

Amir Pakpour

Iran

Santiago Palacios

Spain

Maria Papadakaki

Greece

Malika Patel

South Africa

Nirmala Pathmanathan

Australia

Julietta Patnick

UK

Karl Peltzer

Thailand

Sanja Percac-Lima

USA

Saleem Saaed Qader

Sweden

Atif Rahman

UK

Manee Rattanachaiyanont

Thailand

Maria Theresa Redaniel

UK

Leanne Redman

USA

Ward Rinehart

France

Antonio Rizzo

Italy

Gail Robinson

Canada

Stephen Rogerson

Australia

Ian Ross

South Africa

Pagona Roussi

Greece

Briana Rudick

USA

Khlood Salman

USA

Ana Paula Sampaio Carvalho

Portugal

Ingvild Sandøy

Norway

Clea Sarnquist

USA

Rebecca Say

UK

Karin Schenck-Gustafsson

Sweden

Lone Schmidt

Denmark

Janet Seeley

Uganda

Dorit Segal-Engelchin

Israel

Hailemariam Segni

Ethiopia

Polona Selic

Slovenia

Hideaki Senjyu

Japan

Maurizio Serati

Italy

Isabel Serrano

Spain

Ali Sever

UK

Leigh Anne Shafer

Canada

Babar Tasneem Shaikh

Pakistan

Bo Shen

USA

Chwan-Li (Leslie) Shen

USA

Tarek Shokeir

Egypt

John Simister

UK

Melissa Simon

USA

Kuldip Singh

India

Nilanchali Singh

India

Agneta Skoog Svanberg

Sweden

Joaquim Soares

Sweden

Hong Ji Song

South Korea

Sheila Sprague

Canada

Dawn Stacey

Canada

John Stevenson

UK

Petrus Steyn

South Africa

Nada Stotland

USA

Bjørn Heine Strand

Norway

Min-Ying Su

USA

Minoru Sugiura

Japan

Sharon Talboys

USA

Semara Thomas

USA

Viju Thomas

South Africa

Jane Thompson

Australia

Rebecca Thomson

Australia

Petra Thorn

Germany

Sarah Tinker

USA

Christiana Titaley

Indonesia

Elena Toffol

Finland

Paola Torelli

Italy

Tibor Tot

Sweden

Maria Trigoni

Greece

Artemis Tsitsika

Greece

Odi' Ugochukwu Joannes Umeora

Nigeria

Holger Unger

Papua New Guinea

Elke Van Hoof

Belgium

Zsuzsanna Varga

Switzerland

Saulo Vasconcelos Rocha

Brazil

Freija Verdoodt

Belgium

Viola Verhoef

Netherlands

Denis Walsh

UK

L. Monique Ward

USA

David Wiseman

USA

Li Ping Wong

Malaysia

Sandy Wurtele

USA

Selwa Yacoub

Iraq

Akira Yamasaki

Japan

Kang Youngmi

South Korea

Desalegn Zegeye

Ethiopia

Berihun Megabiaw Zeleke

Ethiopia

Belen Zorrilla

Spain

## Pre-publication history

The pre-publication history for this paper can be accessed here:

http://www.biomedcentral.com/1472-6874/14/9/prepub

